# Supplementary material for: Land, buildings, and equipment acquisitions in U.S. hospitals: A fifteen-year perspective
Source: PLoS One. 2022 Aug 4;17(8):e0272370. doi: 10.1371/journal.pone.0272370 (PMC9352055; doi:10.1371/journal.pone.0272370)
Supplement: Attachment — Submitted filename: ResponseLetter_R1.docx [file pone.0272370.s001.docx]

June 24, 2022

Natasha McDonald, PhD

Associate Editor

*PLOS ONE*

Dear Dr. McDonald:

We were delighted to receive the opportunity to revise our manuscript, “Land, Buildings, and Equipment Acquisitions in U.S. Hospitals: A Fifteen-Year Perspective.” The comments from you and the reviewer were most constructive and guided the revision.

We have revised the manuscript as per your advice and believe that the manuscript has improved considerably as a result. We have submitted a marked-up version and an unmarked version of the revised manuscript. In the following paragraphs, we summarized the journal requirement and the comments from the reviewer, followed by our responses.

**Journal Requirements:**

*1. Please ensure that your manuscript meets PLOS ONE's style requirements, including those for file naming.*

Thank you for the guidance. We have ensured that our manuscript meets PLOS ONE's style requirements, including those for file naming.

*2. Please review your reference list to ensure that it is complete and correct. If you have cited papers that have been retracted, please include the rationale for doing so in the manuscript text, or remove these references and replace them with relevant current references. Any changes to the reference list should be mentioned in the rebuttal letter that accompanies your revised manuscript. If you need to cite a retracted article, indicate the article’s retracted status in the References list and also include a citation and full reference for the retraction notice.*

Thank you for the guidance. We have reviewed our reference list to ensure it is complete and correct.

Dr. McDonald, we thank you again for giving us this opportunity to improve the manuscript and learn from you and the reviewers. We greatly appreciate your guidance and the reviewer’ suggestions. We hope that our responses have effectively addressed the inquiries from the reviewers.

Sincerely,

Ge Bai, PhD, CPA

Professor of Accounting

Johns Hopkins Carey Business School

Professor of Health Policy and Management

Johns Hopkins Bloomberg School of Public Health

Visiting Scholar

Health Analysis Division, Congressional Budget Office

**Reviewers #1's Comments:**

*This is a very straightforward and informative paper on asset acquisition by US hospitals between 2005 and 2019. The authors present basic data on overall trends and aggregate acquisition per capita by state. The analyses are pretty basic but appropriate for questions asked and for the nature of the data available to the authors. The results are purely descriptive, yet novel considering there’s been very little research in this specific area. The limitations are many, however they are adequately acknowledged. Overall, I think the contribution of the new data is significant and will be of interest to many readers. My only suggestion is that the authors discuss asset depreciation. It appears the data may not be available but the bounds it imposes on the interpretability of the results should be discussed. With the net growth in acquisition in single digits in the recent years (even negative in some cases), what can we say about the current asset stock especially for high-depreciation assets like equipment. At least a mention in the limitations section is warranted.*

Thank you very much for the most valuable feedback. We really appreciate your guidance and suggestions and agree with you that in the previous manuscript, discussion on asset depreciation is lacking. In the revised manuscript, we have followed your suggestions and added the following paragraph to the Discussion on page 5:

This study has several limitations. First, the detailed types of operating assets, such as the characteristics of equipment acquired, are unavailable in the data, which prevented us from conducting more specific asset acquisition analysis or understanding depreciation-related issues. Although our study examines the annual acquisitions and their longitudinal changes, our focus is narrowly confined to these asset-related cash outflows and the results cannot be used to accurately determine the current state of asset stock. This is because the annual disposal and depreciation amounts were not analyzed and, more importantly, the market value information of asset stock is not available. For example, low, zero, or negative growth of acquisition does not necessarily indicate a shrinking asset stock, since the disposal and depreciation might have experienced negative growth and the market value of some assets may have appreciated. It is worth noting the distinction between the growth of acquisition and the acquisition amount—zero acquisition would suggest a shrinking assets stock because in any given year depreciation amount cannot be zero.

Dear Reviewer, we thank you once again for your most valuable comments and appreciate having had this wonderful opportunity to learn from you. We hope that our responses have addressed your comments effectively.
