# Supplementary material for: Land, buildings, and equipment acquisitions in U.S. hospitals: A fifteen-year perspective
Source: PLoS One. 2022 Aug 4;17(8):e0272370. doi: 10.1371/journal.pone.0272370 (PMC9352055; doi:10.1371/journal.pone.0272370)
Supplement: Attachment — Submitted filename: Response to Reviewer.docx [file pone.0272370.s002.docx]

**Journal Requirements:**

*1. Please review your reference list to ensure that it is complete and correct. If you have cited papers that have been retracted, please include the rationale for doing so in the manuscript text, or remove these references and replace them with relevant current references. Any changes to the reference list should be mentioned in the rebuttal letter that accompanies your revised manuscript. If you need to cite a retracted article, indicate the article’s retracted status in the References list and also include a citation and full reference for the retraction notice.*

Thank you for the guidance. We have reviewed our reference list to ensure it is complete and correct.

Dr. Pegoraro, we thank you again for giving us this opportunity to improve the manuscript and learn from you and the reviewers. We greatly appreciate your guidance and the reviewer’ suggestions. We hope that our responses have effectively addressed the inquiries from the reviewers.

Sincerely,

Ge Bai, PhD, CPA

Professor of Accounting

Johns Hopkins Carey Business School

Professor of Health Policy and Management

Johns Hopkins Bloomberg School of Public Health

Visiting Scholar

Health Analysis Division, Congressional Budget Office

**Reviewers #1's Comments:**

*Thanks for adding the text about stock vs flow in the limitations. It appears, through, that the last sentence of the new text seemingly contradicts the earlier part, in saying that zero acquisition necessarily means that stock is declining while just before it talks about market value appreciation. The last sentence could be clarified further or removed.*

Thank you very much for the most valuable feedback. We agree with you that the last sentence of the new text contracts the earlier part. We apologize for not noticing this contradiction in the previous manuscript. In the revised manuscript, we have followed your suggestions and deleted this sentence. The revised paragraph (4^th^ paragraph in the Discussion section on page 5) is as follows:

This study has several limitations. First, the detailed types of operating assets, such as the characteristics of equipment acquired, are unavailable in the data, which prevented us from conducting more specific asset acquisition analysis or understanding depreciation-related issues. Although our study examines the annual acquisitions and their longitudinal changes, our focus is narrowly confined to these asset-related cash outflows and the results cannot be used to accurately determine the current state of asset stock. This is because the annual disposal and depreciation amounts were not analyzed and, more importantly, the market value information of asset stock is not available. For example, low, zero, or negative growth of acquisition does not necessarily indicate a shrinking asset stock, since the disposal and depreciation might have experienced negative growth and the market value of some assets may have appreciated.

Dear Dr. Yakusheva, we thank you once again for your most valuable comments and appreciate having had this wonderful opportunity to learn from you. We hope that our responses have addressed your comments effectively.
